# Risk Factors Associated with Poor Outcome in Patients with Infective Endocarditis: An Italian Single-Center Experience

**DOI:** 10.3390/idr14020026

**Published:** 2022-03-21

**Authors:** Claudio Ucciferri, Antonio Auricchio, Carmine Cutone, Alessandro Di Gasbarro, Jacopo Vecchiet, Katia Falasca

**Affiliations:** 1 Clinic of Infectious Diseases, Department of Medicine and Science of Aging, University “G. d’Annunzio” Chieti-Pescara, 66100 Chieti, Italy; claudio.ucciferri@unimol.it (C.U.); auricchio.antonio@hotmail.it (A.A.); reaverblade@hotmail.it (A.D.G.); jvecchiet@unich.it (J.V.); 2 Department of Medicine and Health Sciences, University of Molise, 86100 Campobasso, Italy; c.cutone1@studenti.unimol.it

**Keywords:** infective endocarditis, procalcitonin, in-hospital mortality, embolization

## Abstract

Background: Nowadays, infective endocarditis (IE) is still burdened by a high mortality. In the absence of an adequate prognostic stratification system, it is important to assess new predictors of poor outcomes. The aim of our study is to evaluate which factors were associated with higher mortality in IE patients. Methods: A retrospective cohort study enrolled patients with an IE diagnosis at the Infectious Diseases Clinic of the University ‘G. D’Annunzio’, Chieti, Italy from January 2013 to December 2019. For each patient, demographic, anamnestic and clinical information, embolic phenomena, laboratory and microbiologic data, treatment, and outcomes were collected and analyzed. A correlation analysis was performed. Results: Sixty-eight patients with EI were studied; among them, the mortality was 17.6%, 20.6%, and 23.5%, intra-hospital, at 1 month from discharge and at 6 months from discharge, respectively. Mortality was significantly correlated with age, estimated glomerular filtration rate, and procalcitonin values when considering either basal values (r = 0.266, *p* = 0.029), or values at 48–72 h from the start of an antibiotic therapy (r = 0.222; *p* < 0.05), cerebral embolization for 6-month mortality (r = 0.284; *p* = 0.019), and inadequate antibiotic therapy (r = 0.232, *p* < 0.05). Conclusions: Procalcitonin values, at EI diagnosis and at 48–72 h after starting antibiotics, are prognostic factors useful for stratifying patient risk, and for setting up a personalized treatment. Of note, cerebral embolization and an inappropriate empirical treatment were associated with a higher mortality in the short- and long-term.

## 1. Introduction

Infective endocarditis (IE) is an inflammation of the endocardium, usually involving heart valves, due to bacterial or, less frequently, fungal infection [1]. Over the years, considerable progresses have been performed in understanding the main risk factors, as well as in the diagnostic and therapeutic approaches for IE. For example, the distinction between native valve endocarditis (NVE) and prosthetic valve endocarditis (PVE), distinguished in early-onset IE and late-onset IE, allowed different etiological agents to be considered. Moreover, empiric antibiotic schemes are often based on the combination of different bactericidal drugs and, traditionally, require a prolonged parenteral administration [2]; however, in the absence of viable choices, a bacteriostatic agent, such as linezolid, can be used for methicillin-resistant Staphylococcus aureus (MRSA) or vancomycin-resistant enterococci (VRE) [3,4,5,6,7,8]. In spite of progresses on EI diagnosis and treatment, in-hospital IE mortality still remains high (17.7%) [9]. Due to the variability of clinical onset, some IE cases are not promptly recognized or adequately treated on time. IE diagnosis can occur in the presence of an acute complication, i.e., embolization, or otherwise after non-specific signs and symptoms, including fever, malaise, chills, asthenia, gastrointestinal symptoms, and anemia. Staphylococcal etiology is predominant in IE, accounting for 32% of total IE cases, and is often associated with an unfavorable antibiotic susceptibility profile, i.e., MRSA [10]. IE still represents a significant public health problem in terms of morbidity and mortality. Only a few studies examined predictive factors for mortality in IE [11,12,13,14,15,16]. The aim of this study was to assess which risk factors were associated with poorer outcomes in IE patients admitted to an infectious disease unit in Central Italy.

## 2. Materials and Methods

This is a single-center cohort study, retrospectively enrolling consecutive patients admitted to an infectious diseases clinic, University ‘G. D’Annunzio’, SS Annunziata Hospital of Chieti, Italy, from January 2012 to December 2018. Medical hospital records were examined for each patient, and data regarding anamnesis, clinics, microbiology, diagnosis and therapy were collected and tabulated. The diagnosis of IE was based on Duke’s modified criteria [2] or MRI/PET-Tc positive for IE [17]. Patients with NVEs and PVEs, of any etiology, were included. Patients with suspected diagnosis of IE on admission, not confirmed during hospitalization, were excluded from the study. Furthermore, intra-cardiac device IE cases were excluded from this study. Inappropriate empiric antibiotic therapy was defined when the empirical therapy did not include at least one in vitro active antibiotic against the isolated microorganism. Statistical analysis: Data were analyzed using SPSS Advanced Statistical software version TM 13. Correlation studies were performed for detecting significant associations between mortality in IE patients (at discharge, 30 days and 6 months) and clinical, anamnestic, laboratory and diagnostic features. A Pearson R value was calculated for each parameter, in order to find a linear correlation with mortality. In all statistical tests, the significance threshold was assumed at *p* ≤ 0.05.

## 3. Results

### Study Population

The study enrolled 68 patients; 46 were male (67.6%), and all were of Caucasian ethnicity, with a mean age (±standard deviation, SD) of 65.5 ± 17.4 years. IE population was divided into patients with PVE (n = 24, 35.3%), and patients with an NVE (n = 44, 64.7%). Among the 24 patients with PVE, 3 (12.5%) and 21 (87.5%) had an early-onset and a late-onset endocarditis, respectively. Baseline characteristics of the 68 IE patients are shown in Table 1. In Table 2, clinical manifestations are reported.

**Figure 1:**
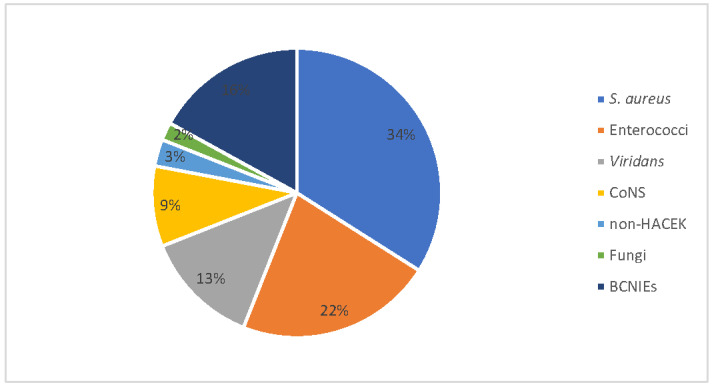
Population of 68 patients with infective endocarditis divided by aetiologic agent. CoNS: coagulase-*negative* staphylococci; HACEK: stands for *Haemophilus* species, *Aggregatibacter* species, *Cardiobacterium hominis*, Eikenella corrodens, and *Kingella* species; BCNIE: blood-culture-negative infective endocarditis.

The organisms most commonly isolated were *S. aureus* (34% of cases), followed by Enterococci (22%), *Streptococcus viridans* (13%) and CoNS (9%). We also recorded one fungal IE case due to *Candida tropicalis*. The number of blood-culture-negative IEs (BCNIEs) was 11 (16.2%) (Figure 1). In terms of valves, the aortic valve was involved in 44%, the mitral valve in 25% and the tricuspid value in 15% of patients; more than one valve was affected in the last 16% of cases. The modified Duke criteria for the IE diagnosis resulted in a 94% diagnosis accuracy, considering definite (n = 47; 69%) and probable (n = 17; 25%) IE cases. Embolic complications occurred in 27 (39.6%) patients, with an extra-cerebral involvement in 20 cases (29.3%). Among these, seven, eight and five patients had an isolated splenic embolization, a pulmonary embolization, and a miscellaneous embolism, respectively (Table 3).

Correlation studies showed that the age of patients (*p* = 0.004) and impaired eGFR values (*p* = 0.004) were correlated to poorer outcomes, either for intra-hospital or for 1- and 6-month mortality. A linear correlation with mortality in IE patients was found between intra-hospital mortality and Pct levels, either for basal Pct or 48–72 h PCT values. Correlations with mortality were also found with a new-onset heart murmur and a discontinuous antibiotic therapy (i.e., interrupted with modified empirical antibiotic scheme). Furthermore, a significant association with an unfavorable 6-month outcome was discovered with neurological signs and symptoms at diagnosis (*p* = 0.019) (Table 4). In our IE population, hypertension, gender, affected valve, WBC values, diabetes and previous prosthetic valve implantation were not significantly correlated with mortality.

## 4. Discussion

Our study confirmed a higher IE mortality rate than that reported in the literature [9]. The analysis of prognostic factors showed a direct proportionality between patient age and poor outcome; eGFR values were inversely correlated with mortality meaning that poor kidney functionality could be used as a predictive factor of a poor outcome, according to the latest European guidelines [2,18]. As already reported in the literature [19], inappropriate empiric antibiotic therapy was associated with a higher in-hospital mortality among our IE patients. Pct values, monitored before (basal Pct, T1) and after (48–72 h from) the beginning of empirical antibiotic therapy, were both associated with an increased intra-hospital mortality. Moreover, as a matter of fact, in our findings, a neurological manifestation was associated with a higher 6-month mortality. On the other hand, our study failed to find a significant correlation between hypertension, gender, affected valve, WBC values, diabetes and previous prosthetic valve implantations with a higher mortality. These findings differ from data reported in the literature [1,2,18,20,21], in which, for example, diabetes has an important prognostic value. As a limitation, the smaller size of our population could have affected the power of our study, which does not allow significant correlations to be found, even in the presence of differences in rates. The findings from our study highlight the critical prognostic role of septic cerebral emboli in IE patients [22,23,24,25]; in particular, the association between cerebral embolic phenomena and 6-month mortality may suggest the important role of the late neurological sequelae in determining a negative outcome. In our study, extra-cerebral embolization was present in 29.4% of IE cases, a percentage higher than previous data from the literature, i.e., 17% according to ICE-PCS [22]. Nevertheless, neither splenic nor pulmonary or renal embolic phenomena were related to a poor intra-hospital outcome; this means that in our experience extra-cerebral embolic involvement could not represent a prognostic factor, as reported in other studies [2,26,27].

In agreement with other studies [16], in our study, the onset of a new heart murmur was associated with increased mortality; indeed, the finding of a new murmur is commonly associated with valve damage and a more-aggressive endocarditis. This evidence reveals the importance of the clinical presentation of the patient at admission and of his/her frequent cardiac auscultation during hospital stay. Procalcitonin still demonstrates its prognostic importance in patients with IE. In the past, other studies evaluated only the poor PCT diagnostic value, which could not be used to confirm a suspected IE [28]. Our study underlined the prognostic role of monitoring PCT values during the hospital stay and follow-up of IE patients. The higher the admission PCT levels (basal Pct, T0), the higher the mortality rate (recorded for in-hospital stay and at follow-up). Higher PCT values at basal time could mean the presence of bacteremia and sepsis at onset, both conditions with a more-severe prognosis. In our findings, the measurement of PCT levels after 48–72 h from the start of the treatment remains a marker of ineffective therapy resulting in an uncontrolled infection, as in the case of inappropriate empirical antibiotic treatment. Diagnostic accuracy, concordant with modified the Duke criteria of our case series, was higher than that reported in ESC guidelines, i.e., 94% vs. 81% [2]; this confirms the validity of our findings, even though, as a major limitation, our data come from a single center, and the size population is relatively limited. Regarding etiology, in our study, S. aureus was the most common organism isolated, as reported by other European studies [29]; however, the prevalence of enterococcal infections was two-fold higher than that reported in the literature, i.e., 22% vs. 11% [1,10], and BCNIE (n = 11, 16.2%). was higher than expected [2,30]. This may reflect, for enterococcal infections, the peculiar ecology of a single center, and for BCNIEs, the previous at-home and in-hospital stay exposure to antibiotics, due to the the paucisymptomatic presentation of the disease. Indeed, 73% of our IE cases presented only a mild fever lasting weeks from admission.

## 5. Conclusions

Our findings, even though from a single center, confirmed the importance of an appropriate risk stratification for IE patients; moreover, they underline the critical role of monitoring procalcitonin levels during hospital stay and follow-up as a prognostic marker of disease progression. High Pct levels are likely the expression of an endocardial and systemic infection not adequately controlled by empirical therapy, and its values must guide clinicians towards an earlier and more aggressive treatment of IE patients. Finally, our findings highlight the importance of following a prompt and appropriate empirical antibiotic treatment, in case of microbiological isolations, by a targeted therapy. This could limit local/systemic IE complications and reduce the rate of adverse outcomes.

This research did not receive any specific grant from funding agencies in the public, commercial, or not-for-profit sectors.

## Figures and Tables

**Table 1:** Baseline characteristics of 68 patients with infective endocarditis.

Age, Year	65.6 ± 17.4
Sex:	
M	46 (67.6%)
F	22 (32.4%)
Drug addiction	10 (14.7%)
Duration of hospitalization, d	23.9 ± 15
Heart Valve:	
• Native valve;	44 (64.7%)
• Mechanical valve;	24 (35.3%)
• Early onset;	3 (12.5%)
• Late onset.	21 (87.5%)
Comorbidities:	
• Diabetes mellitus;	23 (33.8%)
• Hypertension;	42 (61.8%)
• Heart failure;	7 (10.3%)
• Ischemic heart disease;	11 (16.2%)
• Chronic renal failure;	11 (16.2%)
• Immunosuppression.	4 (5.9%)
Hb, g/dL	11.10 ± 2.04
WBC, cell/mm^3^	12,390 ± 6430
PLT, cell/mm^3^	205,560 ± 110,800
INR	1.34 ± 0.49
ERS, mm/h	40.6 ± 35.21
PCR, mg/dL	12.2 ± 14.85
PCT, ng/mL	
- Basal	7.43 ± 20.27
- 48–72 h	3.59 ± 13.10
48–72 h	
eGFR, mL/min/1.73 m^2^	76.7 ± 38.2
Troponin, U/L	0.74 ± 2.60
LDH, U/L	630 ± 470
NT-Pro-BNP, pg/mL	3653.3 ± 848
Ejection fraction, %	59.4 ± 8.01

HB: hemoglobin; WBC: white blood cells; PLT: platelet; INR: international normalized ratio; ERS: erythrocyte sedimentation rate; PCR: C-reactive protein; PCT: procalcitonin; eGFR: glomerular filtration rate; LDH: lactate dehydrogenase; NT-Pro-BNP: N-terminal prohormone of brain natriuretic peptide.

**Table 2:** Clinical manifestations in the cohort of 68 patients with infective endocarditis.

Clinical Manifestation	N (%)
**Fever (with or without chills)**	50 (73.6)
**Cutaneous manifestations**	14 (20.6)
**Focal neurologic deficits**	7 (10.3)
**Asthenia and non-specific symptoms**	17 (25.1)
**New-onset heart murmur**	42 (61.8)

**Table 3:** Embolic complication in 68 patients with infective endocarditis.

Embolization	N (%)
**Splenic**	7 (10.3)
**Pulmonary**	8 (11.7)
**Mixed (splenic ± renal, ± lower limbs, ± mesenteric)**	5 (7.3)
**Cerebral embolization**	7 (10.3)
**Total embolic events**	27 (39.6)

**Table 4:** Significant correlations between discharge, 1-month and 6-month mortality and characteristics of patients.

	Pearson R			*p*-Value		
	Discharge	30 Days	6 Months	Discharge	30 Days	6 Months
**Age**	0.346	0.386	0.386	0.004	0.001	0.001
**eGFR**	−0.343	−0.437	−0.425	0.004	<0.001	<0.001
**Basal PCT**	0.266	0.337	0.339	0.029	0.005	0.005
**48–72 h PCT**	0.222	0.308	0.305	0.049	0.011	0.011
**New-onset heart murmur**	0.311	0.273	0.294	0.010	0.025	0.015
**Non-continued antibiotic therapy**	0.232	0.239	0.283	0.050	0.05	0.019
**Neurological manifestations**	-	-	0.284	-	-	0.019

eGFR: estimated glomerular filtration rate; PCT: procalcitonin.

## Data Availability

Data available upon request.
